# Neuroinflammation and Glial Phenotypic Changes in Alpha-Synucleinopathies

**DOI:** 10.3389/fncel.2019.00263

**Published:** 2019-06-13

**Authors:** Violetta Refolo, Nadia Stefanova

**Affiliations:** Division of Neurobiology, Department of Neurology, Medical University of Innsbruck, Innsbruck, Austria

**Keywords:** microglia, astroglia, alpha-synuclein, neuroinflammation, Parkinson’s disease, multiple system atrophy

## Abstract

The role of neuroinflammation has been increasingly recognized in the field of neurodegenerative diseases. Many studies focusing on the glial cells involved in the inflammatory responses of the brain, namely microglia and astroglia, have over the years pointed out the dynamic and changing behavior of these cells, accompanied by different morphologies and activation forms. This is particularly evident in diseased conditions, where glia react to any shift from homeostasis, acquiring different phenotypes. Particularly for microglia, it has soon become clear that such phenotypes are multiple, as multiple are the functions related to them. Several approaches have over time revealed different facets of microglial phenotypic diversity, and advanced genetic analyses, in recent years, have added new insights into microglial heterogeneity, opening novel scenarios that researchers have just started to explore. Among neurodegenerative diseases, an important section is represented by alpha-synucleinopathies. Here alpha-synuclein accumulates abnormally in the brain and, depending on its pattern of distribution, leads to the development of different clinical conditions. Also for these proteinopathies, neuroinflammation and glial activation have been identified as constant and crucial factors during disease development. In the present review we will address the current literature about glial phenotypic changes with respect to alpha-synucleinopathies, as well as consider the pathophysiological and therapeutic implications of such a dynamic cellular behavior.

## Introduction

Alpha-synucleinopathies are a family of neurodegenerative disorders characterized by the misfolding and accumulation in the brain of alpha-synuclein, a 140-amino-acid protein, encoded by the *SNCA* gene. The physiological form of alpha-synuclein is known to be normally present in neurons, and particularly enriched at pre-synaptic terminals. Its function is still partially unclear, but it seems to be associated with synaptic vesicles’ trafficking during neurotransmitter release (Bellani et al., 2010). The major diseases belonging to the group of alpha-synucleinopathies are Parkinson’s disease (PD), multiple system atrophy (MSA) and dementia with Lewi bodies (DLB) (Spillantini and Goedert, 2000; Goedert et al., 2017). Whereas in PD and DLB alpha-synuclein forms filamentous aggregates in neurons, in form of Lewy bodies and Lewy neurites (Spillantini et al., 1997, 1998a), MSA is mainly characterized by oligodendrocytic inclusions of the protein, called glial cytoplasmic inclusions (GCIs), representing the hallmark of the disease (Papp et al., 1989; Spillantini et al., 1998b; Wakabayashi et al., 1998; Wenning and Jellinger, 2005; Jellinger and Lantos, 2010). PD is one of the most common neurodegenerative diseases, with clinical presentations including bradykinesia, rigidity, resting tremor and gait instability (Gelb et al., 1999). Its main pathological features are loss of dopaminergic neurons in the substantia nigra pars compacta (SNc) and widespread distribution of Lewy bodies and neurites at post-mortem analysis (Forno, 1996; Gelb et al., 1999). Such alpha-synuclein pathology is hypothesized to slowly spread from medulla oblongata and olfactory system to brainstem, limbic system and finally neocortex (Halliday et al., 2011). On the other hand, MSA is mainly characterized by parkinsonism, autonomic failure and cerebellar ataxia in various combinations. GCIs are distributed throughout the brain, and the disease can be subdivided into a parkinsonian (MSA-P) and a cerebellar variant (MSA-C), depending on its main clinical and pathological presentation (striatonigral degeneration (SND) or olivopontocerebellar atrophy (OPCA), respectively) (Fanciulli and Wenning, 2015; Krismer and Wenning, 2017). Finally, DLB is, after Alzheimer disease (AD), the second most frequent cause of dementia in elderly people. It is characterized by visual hallucinations, fluctuating consciousness and parkinsonism (McKeith et al., 2005), with abundant cortical Lewy bodies and various levels of AD-related pathology (Trojanowski and Lee, 1998; Spillantini and Goedert, 2000). Beyond the details that distinguish the single alpha-synucleinopathies, the common picture of alpha-synuclein pathology, neurodegeneration and atrophy, in various degrees, is accompanied by neuroinflammation and glial reactivity, which represent another constant finding in these conditions. In line with findings in other neurodegenerative diseases (Akiyama et al., 2000; Henkel et al., 2004; Mrak and Griffin, 2005; Liu and Wang, 2017), there is cumulative evidence of the participation of neuroinflammatory processes in the development and progression of the pathology for PD and MSA (Gerhard et al., 2003; Wang Q. et al., 2015). As far as DLB is concerned, reports of neuroinflammation and gliosis exist (Mackenzie, 2000; Terada et al., 2000; Surendranathan et al., 2018), but are much less numerous and detailed than the ones addressing other diseases, leaving room for much progress in the field. An increasing number of studies points toward different forms of neuroinflammatory responses, linked to multiple glial phenotypes. From a starting idea of cells broadly acting in a positive or detrimental way on the diseased brain (Cherry et al., 2014), a far more multifaceted picture has emerged over the years. Not only the specific pathology, but also other variables, such as disease stage, brain region and aging process, can indeed influence the glial response to the surrounding stimuli (Grabert et al., 2016; Refolo et al., 2018). Thereby, we will here review the glial phenotypic changes related to neuroinflammatory responses and neurodegeneration in alpha-synucleinopathies, with a particular focus on PD and MSA.

## Glial Cells Involved in Neuroinflammation

The term neuroinflammation (that is, inflammation taking place in the central nervous system (CNS)) comprises a number of events meant to tackle possible or actual threats for the brain. In other words, every time the CNS is faced with infectious agents, traumatic injuries or other unknown elements that might cause a disruption of its homeostasis, it will protect itself by initiation of a series of actions aiming at the elimination of the pathogenic factor. Main actors in this scenario are astroglia and microglia (Kreutzberg, 1995; O’Callaghan and Sriram, 2005). Upon activation, also called “gliosis,” these cells get involved in the production of (and, at the same time, response to) inflammatory cytokines and chemokines, which maintain and enhance the inflammatory condition.

Microglia, in particular, get increasingly highlighted as key players in these processes. Nevertheless, the spectrum of their tasks extends beyond this. Franz Nissl was the first to describe these cells in the end of the nineteenth century, defining them as rod cells (originally “Stabchenzellen”), capable of proliferation, motility and phagocytosis. In 1846, Rudolf Virchow first talked about neuroglia as a kind of glue keeping neurons together, and it became clear only later that these neuroglia were in fact composed of three different cell types, namely oligodendroglia, astroglia and microglia. It was only in 1919 that Pio del Rio-Hortega, a student of the Spanish neuroanatomist Santiago Ramon y Cajal, could distinguish microglia from the other glial cells based on their morphological and functional characteristics (for the English translation of del Rio-Hortega’s four papers where microglia’s description can be found, published in 1919, please refer to Sierra et al., 2016). Firstly believed to have a neuroectodermal origin, it took quite a long time before it was accepted that microglia have a myeloid origin, like macrophages (Perry et al., 1985; Akiyama and McGeer, 1990; Mckercher et al., 1996; Beers et al., 2006). After this finding, new debates opened about the exact origin of their progenitors. Indeed, it was first believed that microglia could be derived from blood-circulating monocytes, hence having a bone marrow origin. However, increasing evidence emerged over time about a separated embryonic origin of microglia. Nowadays it is accepted that microglia originate in the yolk sac and seed the rudimental brain during early phases of fetal development (Takahashi et al., 1989; Takahashi and Naito, 1993; Alliot et al., 1999; Rezaie et al., 2005; Monier et al., 2007; Prinz et al., 2017). Microglia are extremely dynamic cells. Besides their inflammatory action, it has been shown that they are very important in the maintenance of the homeostasis of the brain in healthy conditions, and play significant roles in neural development and plasticity. Microglia represent between 5 and 20% of all glial cells, and 10% of all the cells populating the mammal brain (Lawson et al., 1990; Katsumoto et al., 2014). Interestingly, it seems that the density of microglia differs between brain regions, with highest levels found in the SN (Yang et al., 2013). One of the main features these cells present in homeostatic conditions is their surveillance activity in the brain. It has been shown, also by *in vivo* imaging, that they stretch out and retract continuously their processes in a highly dynamic way, in order to scan the surrounding microenvironment (Nimmerjahn et al., 2005). If, during their surveillance activity, they come across any kind of risk factor, they get activated and start a series of actions meant to get rid of it, as we will discuss later in more detail. Furthermore, they express specific surface molecules and release soluble factors that influence neuronal and astrocytic function (Kettenmann et al., 2011), and are able to clear debris and aggregated proteins (Lee et al., 2010c). In an earlier phase, during fetal development, they assist neurogenesis guiding the formation of prenatal circuits (Squarzoni et al., 2015) and phagocytosing apoptotic cells (Fourgeaud et al., 2016). In post-natal life, microglia continue helping the maintenance of functional neuronal circuits by synaptic pruning. For instance, studies using *in vivo* imaging and high-resolution electron microscopy in mice have shown that microglia get in contact with dendritic spines through their processes, suggesting an active role of these cells in synaptic remodeling (Wake et al., 2009; Tremblay et al., 2010). The awareness of such a role for microglia in the regulation of neuronal networks, in particular during pre-natal and early post-natal phases, has soon prompted the possibility of their involvement in autism spectrum disorders and other neurodevelopmental disorders, such as schizophrenia, and increasing evidence is supporting this idea (Morgan et al., 2010; Tetreault et al., 2012; Sekar et al., 2016; Howes and McCutcheon, 2017). Interestingly, it has been proposed that early synaptic alterations might take place also in alpha-synucleinopathies, as recently shown in the striatum of a PD mouse model (Giordano et al., 2018). These and other observations raise the possibility for glial involvement also in such alpha-synuclein-related events (Aono et al., 2017). This is, for instance, suggested by a study showing restoration of striatal synaptic plasticity in PD rats in association with the reduction of micro- and astrogliosis (Cacace et al., 2017; Ghiglieri et al., 2018). Finally, work by Hagemeyer et al. (2017) suggests that microglia might also contribute to development and homeostasis of oligodendrocyte precursor cells and to myelinogenesis in adulthood.

Also astroglia give an important contribution to neuroinflammation, besides performing many other fundamental functions for the maintenance and homeostasis of the CNS. After Virchow’s proposed concept of neuroglia, the first description of an astrocyte came from Deiters (1865), even though the term “astrocyte” was introduced only later on by Lenhossék (1895) to describe a star-shaped subtype of parenchymal glia. More details about astrocytic morphology and diversity started to be unraveled by Ramon y Cajal and del Rio-Hortega between the end of the nineteenth and the beginning of the twentieth century (Sierra et al., 2016), and, since then, a crescendo of knowledge about these cells has been accumulated. Astrocytes represent the most abundant cell type in the brain. They derive from neuroepithelial radial glia (Kriegstein and Alvarez-Buylla, 2009), although only a portion of astrocytes originates from these precursors during embryonic development; the remaining mature astroglia form during post-natal life by symmetric division of already existing ones (Ge et al., 2012). The actions of astrocytes in the healthy brain are very diverse and key to its proper function. For instance, these cells, and in particular their “endfeet,” are part of the so called neurovascular unit of the blood brain barrier (BBB), thus representing the link between neurons and blood vessels in the brain, and contributing to the great selectivity of this interface between CNS and peripheral tissues (Abbott et al., 2006; Noell et al., 2011). In relation to this, they have also been shown to be involved in the recently discovered “glymphatic system” of the brain. This, comparably to what the lymphatic system does in the rest of the body, allows the elimination of the brain’s waste from the CNS. The astrocytic aquaporin-4 (AQP4), in particular, enables the movement of subarachnoid CSF into the interstitial space, so that CSF can be exchanged with interstitial fluid, and the latter gets eventually driven toward the lymphatic nodes (Iliff et al., 2012; Nedergaard, 2013; Xie et al., 2013). This mechanism might be important also for the clearance of potentially pathogenic proteins, as shown for amyloid-β (Xie et al., 2013). Beside this, astrocytes are fundamental regulators of homeostasis in the brain’s parenchyma. They control ion concentrations in the extracellular space (in particular K^+^), in order to maintain ideal conditions for neuronal excitability and signaling (Song and Gunnarson, 2012; Bellot-Saez et al., 2017). They as well monitor the levels of several neurotransmitters (such as glutamate), which is of paramount importance for their turnover, and to prevent excitotoxicity (Rothstein et al., 1996). Furthermore, they provide an important metabolic support to neurons, for instance through glycogen storage and lactate production (Brown and Ransom, 2007; Stobart and Anderson, 2013). Astrocytes have also been shown to play a role in synaptogenesis and in supporting neuronal connectivity (Eroglu and Barres, 2010; Allen and Eroglu, 2017).

## Neuroinflammation in Alpha-Synucleinopathies

As emerges from the last paragraph, microglia and astroglia are gaining more and more attention from different research fields, alongside with the awareness of the plethora of functions they carry out already in the healthy brain. Nevertheless, as the focus of this review is their role in neuroinflammation in alpha-synucleinopathies, we will now concentrate on this aspect of their activity.

Microglia are indeed known for representing the primary defense line of the CNS. They are surveilling the brain’s parenchyma and constantly monitoring it with their highly motile processes (Nimmerjahn et al., 2005). Every even slight shift from homeostasis leads these very sensitive cells to react and take on a reactive form; this, however, rather than being a simple passage from one state to the other, is a more complex and dynamic process, in which microglia go through different forms of activation, with changes of their morphological and functional characteristics. The main actions that can be undertaken by activated microglia are the production and release of cytokines, chemokines and reactive oxygen (ROS) and nitrogen species (NOS), contributing to the development of the inflammatory event, as well as the phagocytosis of potentially harmful agents and cellular debris. Antigen presentation is also reported in several cases (Lynch, 2009). All of these activities are theoretically beneficial, as long as they are self-limiting, directed against the proper target and quickly resolving after removal of the threat. Functions such as production of anti-inflammatory cytokines, wound healing and debris clearance then allow microglia to restore a normal, homeostatic condition (Cherry et al., 2014). Problems arise, however, when the pro-inflammatory event gets out of control, for instance because of persistence of the hazardous factor, as happens in alpha-synucleinopathies, and in neurodegenerative diseases in general. The ensuing vicious circle, involving the continuous and progressively amplified release of pro-inflammatory molecules and recruitment of further immune mediators, builds up a chronic inflammatory state, leading to toxicity and damage of the surrounding cellular environment. This seems to happen at the cost of the anti-inflammatory, protective functions of microglia, which are at this point not able to keep up with their detrimental effects anymore. It is still a matter of debate whether microglia and neuroinflammation are cause or consequence of the pathological events taking place in neurodegenerative diseases, but there is by now little doubt that they play a role during their progression. As of alpha-synucleinopathies, PET imaging studies have shown the presence of activated microglia in the brains of both PD (Gerhard et al., 2006) and MSA patients (Gerhard et al., 2003), despite some controversies (Ghadery et al., 2017), and increased microglial numbers have been detected by immunohistochemistry in post-mortem PD (McGeer et al., 1988; Imamura et al., 2003; Doorn et al., 2014) and MSA brain tissue (Ishizawa et al., 2004; Salvesen et al., 2015, 2017; Nykjaer et al., 2017). Genetic studies have identified several loci connected to neuroinflammation and microglial activation as risk factors for PD (Hamza et al., 2010; Holmans et al., 2013) and MSA (Nishimura et al., 2002; Infante et al., 2005; Ogaki et al., 2018). In experimental settings, it has been shown that the injection of alpha-synuclein into the SN of rats and mice leads to microgliosis, suggesting that this protein might represent a direct initiator of neuroinflammation (Wilms et al., 2009; Couch et al., 2011). The triggered neuroinflammation, in turn, seems to play a major part in neurodegeneration. It has been shown, for example, that alpha-synuclein-activated microglia enhance neuronal loss *in vitro* (Zhang et al., 2005). Furthermore, through anti-inflammatory agents such as minocycline it has been observed that such interventions have a neuroprotective effect in animal models (Du et al., 2001; Wu et al., 2002), at least if carried out before full-blown pathology is reached (Stefanova et al., 2007), thus suggesting a crucial role for microglial activation, particularly during early stages of the disease.

Astrocytes provide an important contribution during neuroinflammatory responses as well. These cells are able, besides their other multiple functions, to become reactive and work as immune mediators in the brain when elicited by proper stimuli. This includes the recognition of hazard signals, the production and release of cytokines and chemokines and the setting up of a so called glial scar (Sofroniew and Vinters, 2010). The latter is a formation principally made of reactive astrocytes, but also microglia and extracellular matrix, which has the main role of limiting the damage caused by a brain’s lesion, through a sort of sealing effect. If on the one hand this represents the main beneficial effect of the scar, on the other hand there is also production of pro-inflammatory and neurotoxic mediators at its level. Furthermore, until recently it was common belief that glial scars inhibit axon regrowth beyond the scar itself, hampering regeneration (Windle et al., 1952; Silver and Miller, 2004). However, it has been highlighted that positive and detrimental effects might be time dependent, with the first ones appearing immediately after injury and the latter following later on (Rolls et al., 2009), and that glial scar formation might be even crucial for axon regeneration (Anderson et al., 2016). This again points toward the versatility of glial cells and their functions. Astrogliosis has long been described in MSA (Schwarz et al., 1996; Song et al., 2009), with reactive astrocytes observed in striatonigral and olivopontocerebellar structures, the degree of astrogliosis paralleling the neurodegenerative process (Ozawa et al., 2004) and increasing astrogliosis in proximity to GCIs (Radford et al., 2015). In PD this neuropathological feature has often been reported as being less prominent than in MSA (Mirza et al., 2000; Tong et al., 2015), until a role for astrocytes has been recognized in PD pathogenesis too (Durrenberger et al., 2009; Halliday and Stevens, 2011; Booth et al., 2017). Also for MSA there are actually some controversies in this regard, or at least indications for regional differences in astroglial responses. Whereas Salvesen et al. (2015, 2017), for instance, found increased astrocytic numbers in several brain regions, Nykjaer et al. (2017) did not see any significant difference in the number of astroglia within the white matter of MSA cases, with respect to healthy controls. As a side note, one should always be cautious when comparing studies using different parameters to assess microglial or astroglial activation, since increases or decreases in cell counts do not always parallel phenotypic or genotypic changes, which might be better indicators of these cells’ reactivity. Several genetic risk factors for PD, such as DJ-1, parkin and PINK-1, have been implicated in astrocytic function (Hayashi et al., 2000; Bandopadhyay et al., 2004; Neumann et al., 2004; Solano et al., 2008; Choi et al., 2013, 2016; Kim et al., 2016), suggesting a role for astrocytes in PD pathogenesis. Alpha-synuclein, besides accumulating in neurons, has been found in astrocytes in PD as well. It is hypothesized that the protein might be released by the neurons and taken up by astroglial cells (Braak et al., 2007; Halliday and Stevens, 2011). Such a transfer has been indeed observed in *in vitro* and *in vivo* experimental settings, accompanied by the induction of a pro-inflammatory environment (Lee et al., 2010b). Furthermore, it has been shown that cultured astrocytes take up oligomeric alpha-synuclein, however, an excess of it can lead to mitochondrial damage and impaired lysosomal degradation, with consequent accumulation of the protein (Lindström et al., 2017). As of MSA, whereas Song et al. (2009) did not observe any major alpha-synuclein accumulation in astroglial cells, phosphorylated alpha-synuclein aggregates were described in astrocytes located at subpial and periventricular level in another study (Nakamura et al., 2016). Moreover, a mouse model with A53T alpha-synuclein overexpression in astrocytes showed impaired astroglial physiological activity, together with astrogliosis and consequent microgliosis and neurodegeneration (Gu et al., 2010). This study highlighted, among other things, the importance of the crosstalk between microglia and astroglia in pathological settings, pointing, in this case, toward the astrocyte-induced, secondary microglial activation as the main culprit for the neuronal loss observed in the mouse model. Nevertheless, it has also been recently shown that activated microglia are able to induce reactivity of astrocytes, especially toward a pro-inflammatory, neurotoxic phenotype (Liddelow et al., 2017). Altogether, these data seem to indicate that the interaction between glial cells might be more complex and diverse than previously thought, and absolutely worth further in-depth studies.

## Snapshots of Glial Neuroinflammatory Responses to Alpha-Synuclein

**FIGURE 1:**
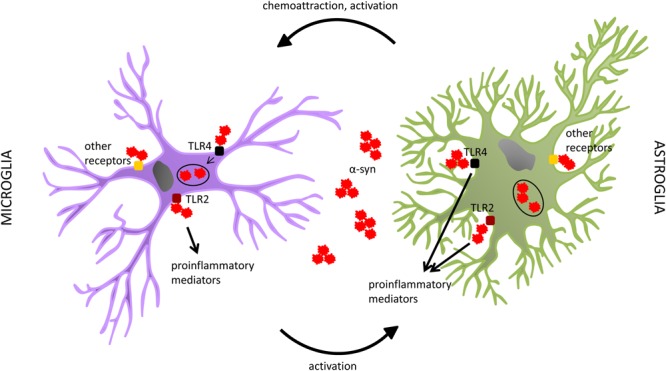
Both microglia and astrocytes have been shown to interact with alpha-synuclein. Among the different receptors suggested to play a role in such interaction, a special focus has been put on TLR4 and TLR2. In microglia, TLR4 seems to be involved in alpha-synuclein clearance, whereas TLR2 rather participates in the production of pro-inflammatory mediators. In astroglia, both receptors have been reported to play a role in the pro-inflammatory response to alpha-synuclein, with no proof of an involvement of TLR4 in the uptake of the protein by this cell type. Both microglia and astroglia can internalize alpha-synuclein. It seems however that, despite their capability to also degrade it in normal conditions, this process might get impaired in alpha-synucleinopathies, probably due to the overload of pathogenic alpha-synuclein, with consequent accumulation of the protein in glial cells. This might lead to their dysfunction and an enhanced neurotoxic profile. Other receptors, and related pathways, have also been associated with glial responses to alpha-synuclein. Further studies are warranted to highlight alternative mechanisms involved in these processes. The interplay between glial cells seems to be relevant as well. Astrocytes, through the release of specific sets of chemokines and cytokines, are able to attract microglia and influence their activation state. On the other hand, recent evidence indicates that also microglia can induce specific astroglial profiles, which can actively affect the pathology progression.

As introduced in the last paragraph, alpha-synuclein definitely exerts an effect on the innate immune system, eliciting the reactivity of glial cells in the brain. Different mechanisms and pathways have started to be unraveled, showing distinct cellular responses. For instance, through binding to CD36, monomeric alpha-synuclein can lead to the production of TNF-alpha and other pro-inflammatory mediators, as well as oxidative stress, by a cascade involving Erk phosphorylation (Su et al., 2008). The same was shown also for mutant alpha-synuclein (Su et al., 2009). A pro-inflammatory microglial response has been shown to be elicited also through FcγR-mediated phagocytosis of aggregated alpha-synuclein (Cao et al., 2012). Further, oligomeric alpha-synuclein interaction with CD11b has been reported to induce activation of the NADPH oxidase NOX2 and consequent toxic effects through production of ROS (Zhang et al., 2005; Wang S. et al., 2015). Similarly, induction of oxidative stress was observed after interaction of oligomeric alpha-synuclein with the purinergic receptor P2X7 (Jiang et al., 2015). Toll-like receptors (TLRs) represent another important class of mediators of microglial reactivity. Among the different existing types of these pattern-recognition receptors (PRR), TLR4 and TLR2 are the ones reported to be most prominently involved in the response to alpha-synuclein. Specifically, TLR4 has been shown to play an important role in the clearance of different forms of alpha-synuclein by microglia, thus providing a neuroprotective effect, as demonstrated in a mouse model of MSA and in *in vitro* settings (Stefanova et al., 2011; Fellner et al., 2013; Venezia et al., 2017). On the other hand, it has been demonstrated by different approaches that TLR2 specifically responds to oligomeric alpha-synuclein with a pro-inflammatory, neurotoxic cascade of events (Kim et al., 2013; Figure 1).

The role of these TLRs has been investigated also in astrocytes. In contrast to what had been observed for microglia, Rannikko et al. (2015) reported that absence of TLR4 did not have any effect on alpha-synuclein uptake by astroglial cells, whereas it decreased their pro-inflammatory responses. Similar findings had been previously reported by Fellner et al., who had also found c-terminally truncated alpha-synuclein as the most potent inducer of glial neurotoxic behavior (Fellner et al., 2013). As to TLR2, Kim et al. (2018) showed that exposure to an anti-TLR2 treatment led to amelioration of pathology and symptoms, with reduced accumulation of alpha-synuclein in neurons and astroglia, in an alpha-synucleinopathy transgenic mouse model (Figure 1). Such effects were reported to be mediated by blockage of alpha-synuclein transmission among neurons, and from neurons to astrocytes (Kim et al., 2018). Such a neuron-to-astrocyte alpha-synuclein transfer had already been previously observed, together with a shift toward an astroglial pro-inflammatory profile (Lee et al., 2010b). In this context, also the idea of astroglia as intermediate players in the activation of microglia during alpha-synuclein pathology arose; astrocytes can indeed release microglia-attractant chemokines, and directly influence their reactivity state through the cytokines they produce (Lee et al., 2010a).

These and other non-reported mechanisms indicate a complexity in glial neuroinflammatory responses (in alpha-synucleinopathies, but not only) which is still mostly unexplored. The tools available until recently allowed us to catch mainly separate processes, like in single snapshots, with little chance of putting the pieces together and looking at the complete picture, in a scenario suggesting multiple functions and ways of action for diverse cellular subtypes. Nevertheless, different methods have helped us over time to discover more about the different facets of glial reactivity and the role of their interplay.

## Microglial Heterogeneity and Phenotypic Changes

Microglia, the innate immune defenders of the CNS, are among the most resourceful cells of our body, being able to perform a myriad of different tasks in health and disease. By doing so they not only contribute to shaping brain architecture and support the development and maintenance of a robust cerebral environment, but they also directly take on and lead the response to homeostasis dysregulations, pathogen attacks and every kind of pathological changes that might occur in the brain. This diversity in functions suggests a great versatility of microglial cells. Already del Rio-Hortega, when first describing microglia, had noticed that they were characterized by a significant degree of heterogeneity even in their normal state, differentiating, for example, between monopolar and bipolar microglia, as well as different types of multipolar microglia, based on the appearance of their processes, such as multipolar cells with branched expansions and multipolar with spiny appendages. He was able to recognize changes in microglial morphology in pathological conditions as well, with increases in dendritic and somatic volume, and sometimes also in cell numbers. He also thoroughly described another type of microglia, the so called “rod cells,” named after their peculiar elongated shape. The characterization of each type of cell was also accompanied by incredibly precise drawings, which del Rio-Hortega was used to make by exactly reproducing what he observed at the microscope. Furthermore, he first recognized microglial functions such as their phagocytic capacity and high motility during pathology (Sierra et al., 2016). Del Rio-Hortega’s meticulous study of microglial features and differences might nowadays surprise for its accuracy and the way it presented, and almost anticipated, details that we are today able to visualize and appreciate with far more advanced techniques than the ones used at his time. Nonetheless, these precious notions remained long neglected, in line with the rejection, by the majority of the academic world of that time, of his idea of the existence of different glial cell types. It took many decades before microglia started again to attract the attention of researchers (Blinzinger and Kreutzberg, 1968; Sierra et al., 2016).

In more recent years, many studies have been undertaken to better understand the diverse physiological and functional characteristics of these very intriguing cells. As one of the most straightforward parameters to assess, **microglial morphology and morphological changes** have been extensively analyzed as measures of microglial activation. It was long shown that microglia tend to adopt a more amoeboid shape, starting from a ramified structure, upon activation (Kreutzberg, 1996; Sierra et al., 2016). Based on the awareness of the plastic and dynamic nature of these cells, different methods have been then established in order to better appreciate the gradual activation process and the involved intermediate phenotypes. For instance, Sanchez-Guajardo et al. (2010) have introduced a morphological classification for microglial activation, which distinguishes these cells in four activation subtypes (called A, B, C, and D), in a rat PD model. Through this classification they have been able to differentiate between microglia going from a resting to a completely activated state based on the appearance of cell body, nucleus and processes (namely, from microglia with thin, long processes and small nucleus and cell body, passing through a state with more abundant secondary ramifications, to one with increased volume of soma and nucleus, together with shortening and thickening of the processes, till the final, amoeboid stage of completely activated cells) (Sanchez-Guajardo et al., 2010). The same method was adapted also to a non-human primate model of PD (Barkholt et al., 2012) and to a mouse model of MSA (Refolo et al., 2018), and proved useful to identify time- and region-dependent differences in the distribution of different microglial subtypes in these models of alpha-synucleinopathies. Several other methods were meanwhile developed in order to make the process more objective and, in some cases, automated. Another subdivision in four types of microglia was obtained through a system of hierarchical cluster analysis of 3D morphometric parameters of the cells (Yamada and Jinno, 2013). Other methods to morphologically distinguish microglial subsets, based on mathematical and computational approaches, include fractal analysis (Karperien et al., 2013; Morrison et al., 2017), Sholl analysis (Morrison and Filosa, 2013; Heindl et al., 2018), as well as combinations of statistics and clustering analyses, as described by Davis et al. (2017). Moreover, Verdonk et al. (2016) developed a system for automated assessment of several parameters related to microglial morphology in CX3CR1^GFP/+^ mice, which present with fluorescent microglia. Beyond the practical differences among the applied approaches, all these methodologies share the ability to show the extreme variability of microglia in normal, but even more in pathologic conditions. Deviations from the classical morphologies, and thus further phenotypic changes, have additionally been observed with aging. For instance, in aged human brains dystrophic microglia have been described, showing fragmented cytoplasm, deramified processes and bulbous swellings (Streit, 2004), whereas reduced complexity (Sierra et al., 2007; Damani et al., 2011) and size (Damani et al., 2011; Hefendehl et al., 2014) of processes have been observed in old rodents. This may represent an important matter of future research in the field of neurodegenerative diseases, since the changes microglia undergo with aging might be an additional, significant factor to be taken into account when considering their role and way of contributing to these diseases. Studies showing phagocytosis deficits in aged microglia support this hypothesis (Njie et al., 2012; Solito and Sastre, 2012; Bliederhaeuser et al., 2016), and recent transcriptomic data suggest the existence of an aging-related phenotype of these cells (Olah et al., 2018), with possible differences between mouse and human aged microglia (Galatro et al., 2017). A separate morphological category of microglial cells, already described, as mentioned before, by del Rio-Hortega, is that of rod-shaped microglia. The existence of these particular cells was actually already reported in 1899 by Franz Nissl, who defined “*Stäbchenzellen”* cells with an elongated cell body, generally located close to neighboring neurons (Nissl, 1899) and often forming “trains” of aligned cells. Described in different neurological conditions (Wierzba-Bobrowicz et al., 2002; Lambertsen et al., 2011; Ziebell et al., 2012), they have been shown to be induced also by alpha-synuclein (Zhang et al., 2005). Although rod-type microglia have been suggested to play a role in synaptic reorganization after injury (Ziebell et al., 2012; Au and Ma, 2017), their exact properties remain elusive, both in alpha-synucleinopathies and in other diseases. Finally, a very recently defined new phenotype of microglia is the “dark microglia,” so called because of their electron-dense cytoplasm and nucleoplasm when analyzed through transmission electron microscopy. These cells are mainly associated with pathological conditions and have been shown to be actively phagocytic, engulfing in particular synaptic elements (Bisht et al., 2016). So far, studies assessing the presence of dark microglia in alpha-synucleinopathies are still lacking.

Even though these and other morphological analyses have been and continue proving fundamental for the understanding of microglial multiple facets, they are generally *per se* lacking substantial information about the **functional correlates** to the described cell subsets. The easiest ways to address this issue generally include the additional use of microglial markers associated with specific functions (such as CD68 for phagocytic activity or MHCII for antigen presentation) or a detailed description of their effects on the surrounding microenvironment, with consequent assumption of the role they might play in such a context. As these approaches are limited and somewhat speculative, a need emerged quite soon for more robust ways to characterize microglial functional phenotypes. The most popular functional classification of microglia is the M1/M2 activation profile, which has been dominating the literature for several years. This classification originally relied on the need for a conceptual simplification of microglia’s spectrum of actions in neuroinflammatory conditions, as well as on the observation of their ability to broadly act either in a beneficial or a detrimental way in the diseased brain. From here the idea to divide microglia in a pro-inflammatory, “classically” activated subset (M1) and in an anti-inflammatory, “alternatively” activated one (M2). This nomenclature was adopted from the M1/M2 distinction originally introduced for macrophages, based on the differential profile they developed under Th1 or Th2 cell specific cytokine stimulation (Nathan et al., 1983; Stein et al., 1992; Doyle et al., 1994; Mills et al., 2000). M1 microglia are generally described as active producers of pro-inflammatory mediators, such as TNFα, IL-1β, IL-6, MIP-1α, ROS and NOS, through which they exert their detrimental effects over prolonged inflammatory periods. On the other hand, M2 microglia are reported to release anti-inflammatory mediators, such as IL-10 and IL-4, and upregulate markers like Arg1 and CD206, being involved in downregulation of the inflammatory process and restoration of a homeostatic condition (Boche et al., 2013; Cherry et al., 2014). However, the availability of more advanced technologies, and their implementation for the study of microglia, has in recent years further broadened our understanding of these cells’ changeable nature and multiple functional subsets, as we will shortly discuss. Even though the M1/M2 classification is still often used or referred to by many studies, there is meanwhile consensus among microglia experts that this is an excessive oversimplification of their varied character, not appropriate anymore to reflect their complexity, and that it should therefore be replaced by more modern concepts (Ransohoff, 2016; Ajami et al., 2018).

Novel, selective **genetic analyses** do indeed represent the new gold standard to get insights into glial multiplicity; these very modern technologies allow in-depth analysis of the cells’ genetic identity and thus clustering in genetic/functional groups that would have never been possible before. In the last few years, several studies have taken advantage of such analyses to unravel new facets of microglial heterogeneous behavior in different conditions. Grabert et al. (2016) were able to confirm regional microglial differences through RNA extraction of microglia isolated from different mouse brain regions. Furthermore, by doing so in mice sacrificed at different ages, they also showed differential changes along the aging process in the analyzed brain regions, which might represent an underlying mechanism for region-specific vulnerability in some brain areas involved in neurodegenerative processes (Grabert et al., 2016). A similar rationale was used by De Biase et al. (2017) to analyze microglial differences among basal ganglia nuclei, which are affected in PD and MSA. Even among these nuclei they found microglial heterogeneity through transcriptome analysis, further showing that the differences started to appear around the second post-natal week of the mice, apparently induced, and later maintained, by specific local cues (De Biase et al., 2017). Tay et al. (2017) observed transcriptional changes between microglia isolated from the ipsi- and contralateral facial nuclei of mice which had undergone unilateral facial nerve axotomy, and differences were evident also at different time points after the lesion. In the same study they also established a new fluorescence fate mapping system, which allowed them to analyze microglial dynamics in living animals through intravital microscopy via a cranial window (Tay et al., 2017). Finally, transcriptional profiling of microglia isolated from the spinal cord of a mouse model of amyotrophic lateral sclerosis (ALS) aided the identification of a protective type of these cells, which seems to be fundamental for the clearance of the pathogenic protein TDP-43 and the regeneration of the affected motor neurons (Spiller et al., 2018). Interestingly, in most of these studies the authors followed also the morphological variations accompanying microglial genetic changes, showing the willingness to link the newly addressed transcriptomic data with the classic morphological profiles.

Even though these analyses on discrete microglial groups had already represented a huge step further in the comprehension of the genetic background of their heterogeneity, the advent of **single-cell technologies** has led the research in the field to the next level. Considering the repeatedly proven changes that microglia undergo, it was not pointless to expect an even greater variability, which single-cell analyses would be even more sensitive to. To begin with, Mathys et al. (2017) applied single-cell RNA sequencing to microglia isolated from the hippocampus of an AD-like neurodegeneration mouse model, and found distinct subsets, changing with disease stage and degree of neurodegeneration. Using a slightly different approach, that is single-cell mass cytometry, Ajami et al. (2018) demonstrated the presence of different myeloid cell profiles among mouse models of different diseases, whereas Bottcher et al. (2019) confirmed region-specific heterogeneity also for human microglia. Very interestingly, in a work of 2017, Keren-Shaul and colleagues performed single-cell transcriptome analysis of microglia isolated from an AD mouse model and discovered a new microglia type, which they called “DAM” (disease-associated microglia), completely absent in healthy wild-type animals. These cells were reported to be activated in two sequential steps (the first one being Trem2-independent while the second one Trem2-dependent), to be mostly concentrated around amyloid-β (Aβ) plaques and to have phagocytic activity toward Aβ, overall described as having a beneficial effect on AD pathology. Furthermore, they found DAM also in an ALS mouse model (Keren-Shaul et al., 2017). Through the same technology, in a subsequent study Tay et al. (2018) identified a unique microglia population, ensuing specifically in the beginning of the recovery phase in a neurodegeneration model, and these cells showed high transcriptional similarity with the DAM described by Keren-Shaul et al. (2017). Moreover, also in the previously cited work by Mathys et al., one of the microglial subpopulations detected by the authors, mainly appearing in an advanced stage of the disease, shared many genes with DAM (Mathys et al., 2017). In a recent study, Masuda et al. further found disease-specific microglial subsets, in addition to age- and region-specific ones, after analysis of single microglial cells isolated from mouse and human brains (Masuda et al., 2019). These studies show the relevance of such analyses, not only for a more in-depth appreciation of the multiple microglial phenotypes, but, even more importantly, for the discovery of subsets of these cells directly involved in specific mechanisms/disease stages/functions during pathologic processes. The possibility of identifying selected targets, and knowing their exact genetic signature, indeed opens concrete chances for tailored therapeutic interventions. DAM might represent one of microglial subpopulations to be modulated for this purpose. As they have been detected already in AD and ALS models, it is possible that they might play a role also in other neurodegenerative conditions. Both for this and for other candidate microglial subgroups, this kind of studies represents the future also in the field of alpha-synucleinopathies, and, once addressed, will for sure lead to enormous progress in the understanding of microglial contribution to these pathologies.

## Astroglial Heterogeneity and Phenotypic Changes

When looking at what is known about astroglial phenotypic heterogeneity and changes, the situation is quite different if compared to microglia. That the term astrocyte comprises a varied population of cells already in the healthy brain is actually nothing new, as different morphologies, markers, functions and cerebral locations have been long reported. The first distinction that has been made was the one between protoplasmic astrocytes, residing in the gray matter and presenting with a ramified morphology, and fibrous ones, which can be found in the white matter and have long, scarcely ramified processes (Ramon y Cajal, 1909; Sofroniew and Vinters, 2010). In PD, accumulation of alpha-synuclein was observed in protoplasmic astrocytes but not fibrous ones, whereas in MSA fibrous astrocytes where the ones shown to be the most reactive to the concomitant pathology (Song et al., 2009). Specialized astroglia have then been detected, such as Müller glia in the retina and Bergmann glia in the cerebellum, but also velate astrocytes, tanycytes, ependymal glia, marginal glia, perivascular glia (Emsley and Macklis, 2006) and further categories that can be found throughout the literature (Verkhratsky and Nedergaard, 2018). Both inter-regional and intra-regional differences have been highlighted, not only morphologically, but also in terms of functions and protein expression (Garcia-Abreu et al., 1995; Oberheim et al., 2012). In recent years, more advanced technologies have been implemented in the field. For instance, five different astrocytic subpopulations were identified through an intersectional fluorescence-activated cell sorting (FACS)-based method, and subsequent RNA sequencing, in different mouse brain regions (John Lin et al., 2017), whereas an extensive study on the mouse brain at the single-cell level revealed the presence of seven transcriptionally distinct types of astrocytes (Zeisel et al., 2018).

However, phenotypic changes upon astrogliosis are far less known than the ones occurring in microglia, as this research field is still in its infancy. Also for astrocytes, pathological changes in the CNS lead to their activation. Reactive astrocytes are generally recognized for being hypertrophic, upregulating glial fibrillary acidic protein (GFAP, one of the most used astroglial markers) and often for the formation of a glial scar, as already previously discussed in the text (Sofroniew and Vinters, 2010). This view of reactive astrogliosis has however evolved in the last years, driven by new studies focusing on this process. In 2012, by transcriptome analysis of astrocytes isolated from an ischemia and an inflammation mouse model, it was shown that both pathologic conditions elicited reactivity of these cells, but in different ways; whereas the differentially expressed genes after stroke suggested a rather protective phenotype, the ones upregulated upon neuroinflammation indicated potentially detrimental effects (Zamanian et al., 2012). Similarly, “pro-” and “anti-inflammatory” astroglial polarization states were observed also in another work, through *in vitro* and *in vivo* approaches (Jang et al., 2013). Thus, the dichotomy long used for macrophages and microglia started to be considered also for astrocytes. Indeed, in 2017 Liddelow and coworkers introduced the distinction between “A1” and “A2” astrocytes; they defined as A1 those with a detrimental function, showing upregulation of genes involved in the classical complement cascade, and A2 those with a beneficial effect, mainly upregulating genes encoding for neurotrophic factors. This was done in the context of a study where they showed that activated microglia, by production and release of mediators such as TNF, Il-1α and C1q, were the direct initiators of astroglial polarization toward the A1 phenotype. These astrocytes, in turn, took on a neurotoxic profile and caused degeneration of neurons and oligodendrocytes (Liddelow et al., 2017). This, among other findings, confirmed the importance of the crosstalk between microglia and astrocytes, and opened new exciting possibilities for the understanding of glial involvement in pathological processes. In their work, Liddelow et al. (2017) further showed that post-mortem PD brains (as well as those of patients with other neurodegenerative conditions) were positive for A1 markers in regions affected by the disease, thus postulating that these astrocytes might play a role in neurodegeneration. Soon after, Yun et al. demonstrated that, by preventing microglial activation through administration of a glucagon-like peptide-1 receptor (GLP1R) agonist, they were able to hinder astroglial transition toward an A1 phenotype, and this resulted in neuroprotection and preservation of motor functions in two mouse models of PD (Yun et al., 2018). On the other hand, another study showed that microglia can lead to the induction of an astrocytic neuroprotective profile via a pathway involving the P2Y_1_ receptor (Shinozaki et al., 2017). On the basis of what has happened in the microglia field, it remains to be seen whether this A1/A2 classification will prove appropriate for astrocytes, or whether it will need to be further revised. Nevertheless, these studies have been fundamental for offering a new angle to look at astrocytes and glial interactions, and have paved the way for new research targets in the field. Another type of change that has been associated with astroglial involvement in disease is that coming along with aging and cellular senescence. In 2018, Chinta and colleagues demonstrated that paraquat (a neurotoxin which has been associated with increased risk of developing PD) induces astrocytic senescence in PD brains, as well as in experimental models, suggesting that this factor might contribute to PD pathology (Chinta et al., 2018). In line with this, a study analyzing the transcriptome of aging astrocytes, isolated from different mouse brain regions, showed differential age- and region-specific changes, possibly related to specific pathologic mechanisms taking place in aging-associated diseases (Boisvert et al., 2018). Furthermore, Clarke et al. (2018) observed, through a similar approach, the induction of the astrocytic A1 phenotype with aging. Also in this case, microglia were found to be the promoting agent for this shift. Altogether, it is clear that big advances have been made, especially in very recent years, in the understanding of astroglial reactivity and contribution to neurodegenerative processes, such those taking place in alpha-synucleinopathies. The dialog with microglia seems to be of critical importance in this context, and will for sure represent the focus of further exciting research in the near future. Nevertheless, as suggested by the finding of different, molecularly defined astroglial subpopulations already in the healthy brain (John Lin et al., 2017; Zeisel et al., 2018), there is probably still much more to be uncovered about the fine changes that their profiles can undergo in distress conditions, and population and single-cell molecular analyses will also in this case open up many new possibilities.

## Implications for Therapeutic Approaches

To date, PD and MSA are still lacking a cure. Researchers are struggling in the attempt to uncover pathogenic mechanisms and significant contributing factors, in order to find exact targets to address for effective therapeutic options, but every effort so far has failed, particularly when coming to clinical trials. Among the pathogenic candidates, neuroinflammation has gained a relevant position over time, along with the increasing recognition of the role it plays in alpha-synucleinopathies (Zhang et al., 2005; Stefanova et al., 2007; Wilms et al., 2009; Couch et al., 2011). Several treatments, targeting this process at different levels, have been experimented, and in most cases they have proven successful when tested in animal models. One example for this is minocycline, a tetracycline antibiotic showing anti-inflammatory effects, apparently through interference with the release of pro-inflammatory mediators by microglia (Tikka and Koistinaho, 2001; Wu et al., 2002). Although conflicting results emerged from different studies (Chen et al., 2000; Zhu et al., 2002; Smith et al., 2003; Diguet et al., 2004), it is generally accepted that minocycline has the potential for being neuroprotective in various neurological conditions, as has been demonstrated in several occasions (Zhu et al., 2002; Wang et al., 2003; Hunter et al., 2004). It has been tested in PD and MSA animal models as well, showing positive effects in terms of neuroinflammatory responses and neuropathology (Du et al., 2001; He et al., 2001; Wu et al., 2002; Tomás-Camardiel et al., 2004; Stefanova et al., 2007). However, when going into clinical trials, in none of the two diseases minocycline exerted the hoped beneficial effect (NINDS NET-PD Investigators, 2008; Dodel et al., 2010). In MSA patients, it even lowered the levels of activated microglia with respect to the placebo-treated group, but this was not sufficient to ensure also an amelioration of the motor symptoms (Dodel et al., 2010). This is a good example to point out the limitations of most of the treatments targeting neuroinflammation so far. One of the major issues is the timing of treatment initiation. As demonstrated in the experimental models getting minocycline, it seems to be of critical importance to start the administration before onset of the clinical symptoms. When neurodegeneration has already abundantly taken place, which is the case in clinically diagnosed patients, it is apparently too late to target microglial activation, at least with the compounds tested up to now. This is also in line with the demonstrated early onset of neuroinflammatory responses during the development of alpha-synucleinopathies (Stefanova et al., 2007; Marinova-Mutafchieva et al., 2009; Refolo et al., 2018). Early treatment seems to be essential for success.

**FIGURE 2:**
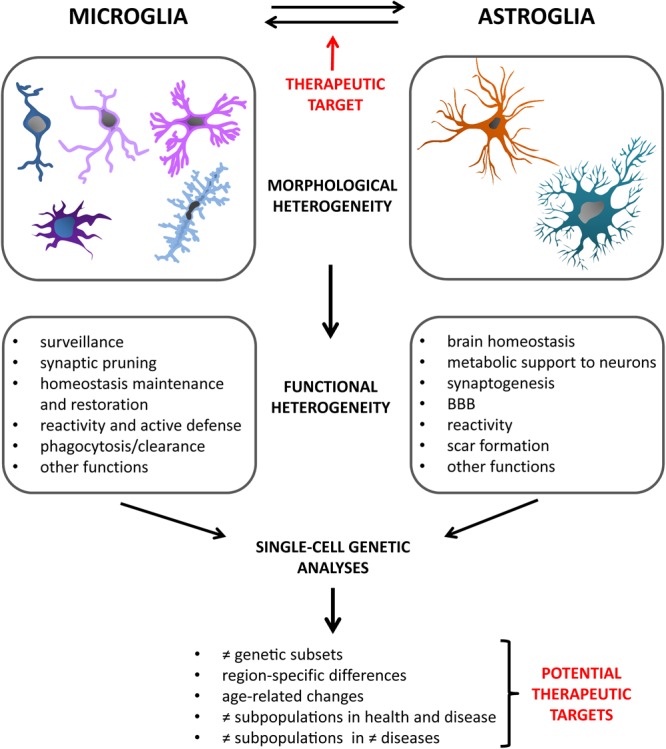
Glial cells are characterized by great heterogeneity. Besides their multiple morphological phenotypes, they present with an impressive variety of functions, both in health and disease. New technologies are progressively allowing us to acquire increasingly in-depth insights into these varied glial profiles. In particular, single-cell genetic analyses have in recent years opened up a new range of possibilities in this regard. They have indeed revealed the existence of different glial subsets, defined on a genetic basis. Moreover, studies carried out considering different variables, such as age, brain regions, healthy or diseased state, have added further facets to the already intricate picture of the diverse glial subtypes. The field of alpha-synucleinopathies will also greatly benefit from studies of this kind in the near future, creating the possibility for novel, targeted therapeutic approaches. Also the microglia-astroglia crosstalk has been recently confirmed as an important way by which glial cells can mutually influence the respective activation profiles and responses to pathology. This might offer another therapeutic target to assess in future studies.

However, another problem might be the specificity of such drugs. Next-generation population and single-cell molecular analyses have opened our eyes on a much higher variability in microglial responses than ever thought. Various subpopulations of microglia have already been described, with region-, age- and disease-specific differences, and the study of this variegation is only in its beginnings (De Biase et al., 2017; Mathys et al., 2017; Ajami et al., 2018; Bottcher et al., 2019; Masuda et al., 2019). In particular, the discovery of DAM, a type of microglia found in association with diseased conditions (Keren-Shaul et al., 2017), has introduced the possibility of specific profiles having specialized functions during pathological processes. In the light of this, it is clear that targeting discrete microglial subpopulations will represent the future for new therapeutic approaches. Indeed, aiming at general microglial pathways, which may be common to different types of them, risks to be counterproductive and even deleterious in the long term. For instance, in case of infection, or any other type of acute event for which a pro-inflammatory intervention by microglia would be of vital importance, such generalized inflammation suppressants could do more harm than good. Knowing exactly which subtypes of cells exert the real, maybe pure detrimental effects, via specialized pathways, will enable us to detect the proper targets to silence, and get a step closer to our goal. Moreover, not only the “bad microglia” can be exploited to improve a pathological state. The DAM described by Keren-Shaul et al. (2017), as well as microglial subpopulations observed in other studies (Spiller et al., 2018; Tay et al., 2018), were recognized as acting in a beneficial way on certain aspects of the pathology, and to ensue during defined stages of the disease. Specifically boosting cells with such a protective profile has therefore the potential to represent another powerful strategy to steer the disease course in a tailored way, for instance by enhancing regenerative effects or the clearance of pathogenic proteins. Combination of such “customized” treatments, precisely dampening or promoting exact, molecularly defined, microglial subtypes, might be the winning move for successful modulation of what is comprised in the broad concept of “microglial activation.” Furthermore, based on the observation of different microglial molecular profiles among models of distinct pathologies (Ajami et al., 2018; Masuda et al., 2019), it is possible that also the subpopulations to be pharmacologically targeted may differ from disease to disease. Further research will be needed to discover such pathology-specific microglial niches and, where appropriate, adjust therapeutic interventions accordingly (Figure 2).

In the regard of treatments targeting neuroinflammation, it has to be recognized that microglial activation is generally meant as the main focus of such approaches. However, several studies have started to highlight the relevant and often neglected role that also astroglial reactivity plays during neuroinflammatory responses, such as those taking place during alpha-synucleinopathies (Durrenberger et al., 2009; Song et al., 2009; Booth et al., 2017). Particularly, in recent years, it has emerged that also astrocytes possess at least a dichotomous behavior, with polarization states showing pro- or anti-inflammatory actions (Liddelow et al., 2017) and apparently offering further chances for therapeutic interventions. As discussed before, also for astrocytes molecular analyses at the single-cell level will help to find out more about their variable nature in response to diseased conditions, and will thus increase the selection of targets for single and combined treatments. Nevertheless, even with a greater awareness of astroglial importance *per se*, microglia are far from being out of the scene. Indeed, many of the most recent studies addressing astrocytic differential involvement in neuroinflammation and pathology have as well shown that microglia are able, through specific cues, to guide astrocytes toward different functional profiles (Liddelow et al., 2017; Shinozaki et al., 2017; Yun et al., 2018). This shows that also the crosstalk between microglia and astrocytes should not be underestimated, but rather further analyzed, in particular in the context of neurodegeneration (Figure 2). A better understanding of the way glial cells interact, also in the light of their multiple phenotypes, specific pathology and disease stage, will be key to the development of proper therapeutic strategies.

## Conclusion

There is by now little doubt that neuroinflammation is a factor at least heavily influencing the pathology development in alpha-synucleinopathies like PD and MSA. Being the big protagonists in this context, microglia, and over time also astrocytes, have been recognized as important elements to be modulated for therapeutic interventions in these diseases, as well as in neurodegenerative conditions in general. Even though the idea of these cells being able to acquire different phenotypes, accompanied by different morphologies and functions, was not new, the advent of modern imaging techniques, followed by advanced molecular analyses, has greatly widened our knowledge about their multiple ways to act in health and react in disease. Even more recently, RNA sequencing and other technologies, carried out at the single-cell level, have shed additional light on glial heterogeneity; they are enabling us to identify various glial subpopulations, differing in a region-, age-, disease- and disease stage-specific way, that we are just starting to appreciate. Further studying glial subpopulations in a context-dependent manner, and at the level of single cells, will be crucial for future identification of specific therapeutic targets; it will indeed allow for therapies aiming at neuroinflammation in an intelligent way. Furthermore, the microglia-astroglia crosstalk, long overlooked and just recently gaining new attention, has also lately demonstrated to be significant for the regulation of neuroinflammatory responses. This might represent an additional target for disease modifying strategies, and will thus need to be further addressed in the future. Glial variability and multiple phenotypes might seem confusing. Nevertheless, this heterogeneity is an invaluable tool for targeted interventions in diseases characterized by prominent neuroinflammatory events, and its understanding will open a whole range of new possibilities for these conditions.

## Author Contributions

VR conceived and wrote the manuscript. NS gave feedback and edited the manuscript.

## Conflict of Interest Statement

The authors declare that the research was conducted in the absence of any commercial or financial relationships that could be construed as a potential conflict of interest.
